# Zic Family Member 2 (ZIC2): a Potential Diagnostic and Prognostic Biomarker for Pan-Cancer

**DOI:** 10.3389/fmolb.2021.631067

**Published:** 2021-02-16

**Authors:** Zhengtong Lv, Lin Qi, Xiheng Hu, Miao Mo, Huichuan Jiang, Benyi Fan, Yuan Li

**Affiliations:** ^1^ Department of Urology, Beijing Hospital, National Center of Gerontology, Institute of Geriatric Medicine, Chinese Academy of Medical Sciences, Beijing, China; ^2^ Graduate School of Peking Union Medical College and Chinese Academy of Medical Sciences, Beijing, China; ^3^ Department of Urology, Xiangya Hospital, Central South University, Changsha, China

**Keywords:** pan-cancer, Zic2, prognosis, TCGA, biomarker

## Abstract

**Background:** As a transcription factor, Zinc finger protein ZIC2 can interact with various DNAs and proteins. Current studies have shown that ZIC2 plays an oncogene role in various cancers. In this study, we systematically characterize the prevalence and predictive value of ZIC2 expression across multiple cancer types.

**Methods:** We mined several public databases, including Oncomine, the Cancer Genome Atlas (TCGA), cBioPortal, Kaplan-Meier Plotter and PrognoScan to evaluated the differentially expressed ZIC2 between tumor samples and normal control samples in pan-cancner, and then explored the association between ZIC2 expression and patient survival, prognosis and clinicopathologic stage. We also analyzed the relationship between tumor mutation burden (TMB), microsatellite instability (MSI), tumor microenvironment, tumor- and immune-related genes and ZIC2 expression. Finally, we explored the potential signaling pathway mechanism through gene set enrichment analysis (GSEA).

**Results:** ZIC2 expression was higher in most cancer tissues compared with adjacent normal tissues. High ZIC2 expression was associated with worse prognosis and a higher clinicopathologic stage. ZIC2 expression was strongly associated with the TMB, MSI, tumor microenvironment and tumor- and immune-related genes. The GSEA revealed that multiple tumor- and immune-related pathways were differentially enriched in ZIC2 high or low expression phenotype.

**Conclusion:** ZIC2 expression may be a potential prognostic molecular biomarker of poor survival in pan-cancer and may act as an oncogene with a strong effect in the processes of tumorigenesis and progression.

## Introduction

Cancer is a major public health problem worldwide and is caused by many factors (Siegel et al., 2020). It is important to identify key tumor-related genes to improve the understanding of cancer initiation, maintenance and progression (Andre et al., 2014). Large sample, high-throughput and multiple cancer data will help to find the key genes of carcinogenesis. Some medical public database, such as Gene Expression Omnibus (GEO) and The Cancer Genome Atlas (TCGA), provided multiple omics data for different cancers, making it possible to conduct pan-cancer studies in different omics (Xiong et al., 2019; ICGC/TCGA Pan-Cancer Analysis of Whole Genomes Consortium, 2020).

There are five genes in Zic family, Zic family member 2 (ZIC2) has C2H2 zinc fingers and can interact with multiple DNA and proteins. ZIC2 plays a role in a variety of human development processes, including neuromyogenesis, skeletal patterning, and left-right axis establishment (Grinberg and Millen, 2005; Ali et al., 2012). Current studies reported that ZIC2 may act as an oncogene in multiple cancers (Chen et al., 2019; Jiang et al., 2019; Liu et al., 2020; Yu et al., 2020). And its overexpression has been found to be related to invasiveness and metastasis in nasopharyngeal, breast, prostate cancer, acute myeloid leukemia, and so on, which sparked our interest in the gene ZIC2. However, the role of ZIC2 in other cancers remains unknown. At present, there is no comprehensive study on the prognostic significance of ZIC2 in pan-cancer.

In this study, we systematically characterize the prevalence and predictive value of ZIC2 expression across multiple cancer types. Meanwhile we also explored the correlation between ZIC2 expression and tumor mutation, immune microenvironment, and various tumor- and immune-related genes. Finally, we explored the biological function and pathways of ZIC2 through Gene Set Enrichment Analysis (GSEA).

## Materials and Methods

### Data Acquisition

The transcriptome data, mutation data and clinicopathological data of 33 kinds of cancer in TCGA were acquired from the UCSC Xena project (http://xena.ucsc.edu). The sample size of 33 malignant tumors and the expression of ZIC2 are summarized in Supplementary Table S1. We also analyzed the Oncomine database (http://www.oncmine.org) to detect the expression difference of ZIC2 in pan-cancer and adjacent normal tissues. To verify the prognostic value of ZIC2, we assessed the Kaplan-Meier Plotter (http://kmplot.com/analysis) and PrognoScan (http://www.prognoscan.org) for verification. These databases collected cancer microarray datasets including GEO, ArrayExpress, European Genome-phenome Archive and other individual laboratory web sites, which can serve as a very good external validation data set for TCGA.

### Survival Analysis

The patients were divided into high-risk group and low-risk group according to the median expression level of ZIC2. The Kaplan-Meier curves (K-M) was created to display the differences in patient survival and univariate Cox regression model was utilized to assess the prognostic significance of ZIC2 expression regarding Overall Survival (OS), Disease-Specific Survival (DSS), Disease-Free Interval (DFI), Progression-Free Interval (PFI), Relapse-Free Survival (RFS) and Distant-Metastasis-Free Survival (DMFS).

### Calculation of TMB and MSI

To calculate the tumor mutation burden (TMB), the number of somatic mutations is divided by the whole length of exons (38 Mb) (Binnewies et al., 2018). To calculate the microsatellite instability (MSI), we used *R* package of PreMSIm to predict MSI from gene expression profile in 33 kinds of cancer (Li et al., 2020).

### Determination of Immune Microenvironment and Immune Cells

We downloaded the R script of ESTIMATE algorithm and calculated the immune scores and stromal scores for each tumor sample, respectively. The 22 immune cell infiltrates (B cells naive, B cells memory, Plasma cells, T cells CD8, T cells CD4 naive, T cells CD4 memory resting, T cells CD4 memory activated, T cells follicular helper, T cells regulatory (Tregs), T cells gamma delta, NK cells resting, NK cells activated, Monocytes, Macrophages M0, Macrophages M1, Macrophages M2, Dendritic cells resting, Dendritic cells activated, Mast cells resting, Mast cells activated, Eosinophils and Neutrophils) were obtained using CIBERSORT (Newman et al., 2015). The analysis tool can use gene expression profile data to estimate the relative abundance of these 22 immune cell types.

### Gene Set Enrichment Analysis (GSEA)

To identify biological function and potential signaling pathway of ZIC2, the functional annotation of Kyoto Encyclopedia of Genes and Genomes (KEGG) was performed.

### Statistical Analysis

All analyses were completed by *R* (version 3.6.1) and *R* packages. The comparisons of ZIC2 expression between tumor and normal tissues were tested by Wilcoxon rank sum test. Pearson correlation coefficient test is used to analyze the correlation. *p* values below 0.05 was considered significant.

## Results

### Expression of ZIC2 in Pan-Cancer

**FIGURE 1:**
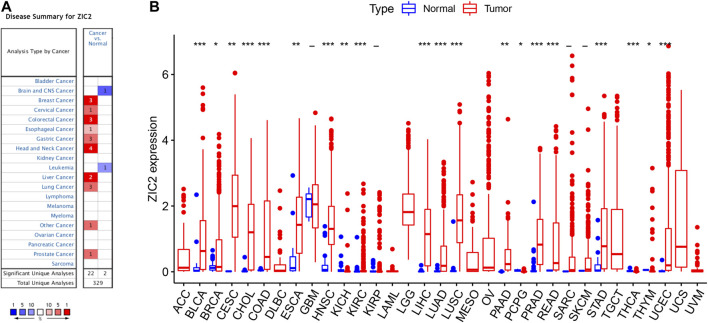
The differential expression of ZIC2 gene in cancer and its corresponding normal tissues in pan-cancer. **(A)** Oncomine dataset. **(B)** TCGA dataset. (***p < 0.001; **p < 0.01; *p < 0.05; -, no statistical difference)

We first used Oncomine to investigate the expression of ZIC2 gene across cancers compared with corresponding normal tissues. Our results suggest that ZIC2 expression is higher in many cancer groups, including the breast, cervical, colorectal, esophageal, gastric, head and neck, liver, lung, prostate and seminoma. Only brain, CNS and leukemia showed low ZIC2 expression (Figure 1A). We also obtained the expression of ZIC2 gene in cancer and normal tissues in 33 cancer samples from the TCGA database. We similarly found that ZIC2 was highly expressed in 20 cancer tissues compared to the corresponding normal tissues (BLCA, BRCA, CESC, CHOL, COAD, ESCA, HNSC, KICH, KIRC, LIHC, LUAD, LUSC, PAAD, PCPG, PRAD, READ, STAD, THCA, THYM and UCEC) (*p* < 0.05). Only four cancers showed no significant difference in expression of ZIC2 (GBM, KIRP, SARC and SKCM) (*p* > 0.05). In the remaining nine types of tumors, the differential expression of ZIC2 could not be known due to the absence of normal tissue control (ACC, DLBC, LAML, LGG, MESO, OV, TGCT, UCS and UVM) (Figure 1B).

### Correlation of ZIC2 Expression and OS

**FIGURE 2:**
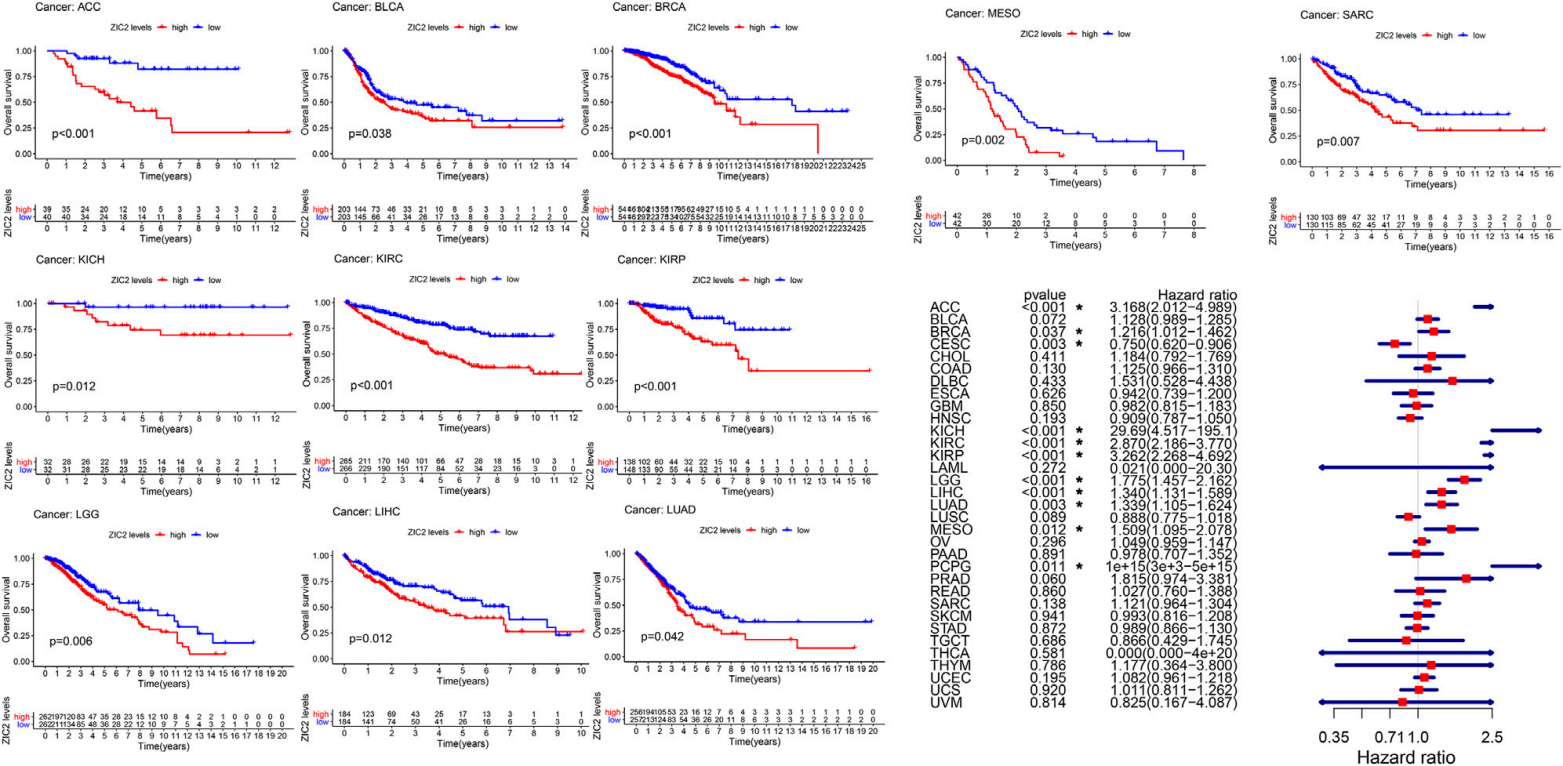
The relationship between ZIC2 expression and OS in 33 tumors. The survival curves were calculated using K-M methods and the forest plots were calculated using univariate Cox regression. (*p < 0.05)

We used gene expression profile data to analyze the relationship between ZIC2 expression and OS in 33 TCGA tumors. K-M curve revealed that high expression of ZIC2 was associated with poor OS time in 11 types of cancer (ACC, BLCA, BRCA, KICH, KIRC, KIRP, LGG, LIHC, LUAD, MESO and SARC). The univariate cox regression also showed that ZIC2 expression was related to OS time in 11 types of cancer (ACC, BRCA, CESC, KICH, KIRC, KIRP, LGG, LIHC, LUAD, MESO, and PCPG). Among them, high ZIC2 expression was all a risk factor in 10 tumors, and was only a protective factor in CESC (Figure 2).

### Correlation of ZIC2 Expression and DSS

**FIGURE 3:**
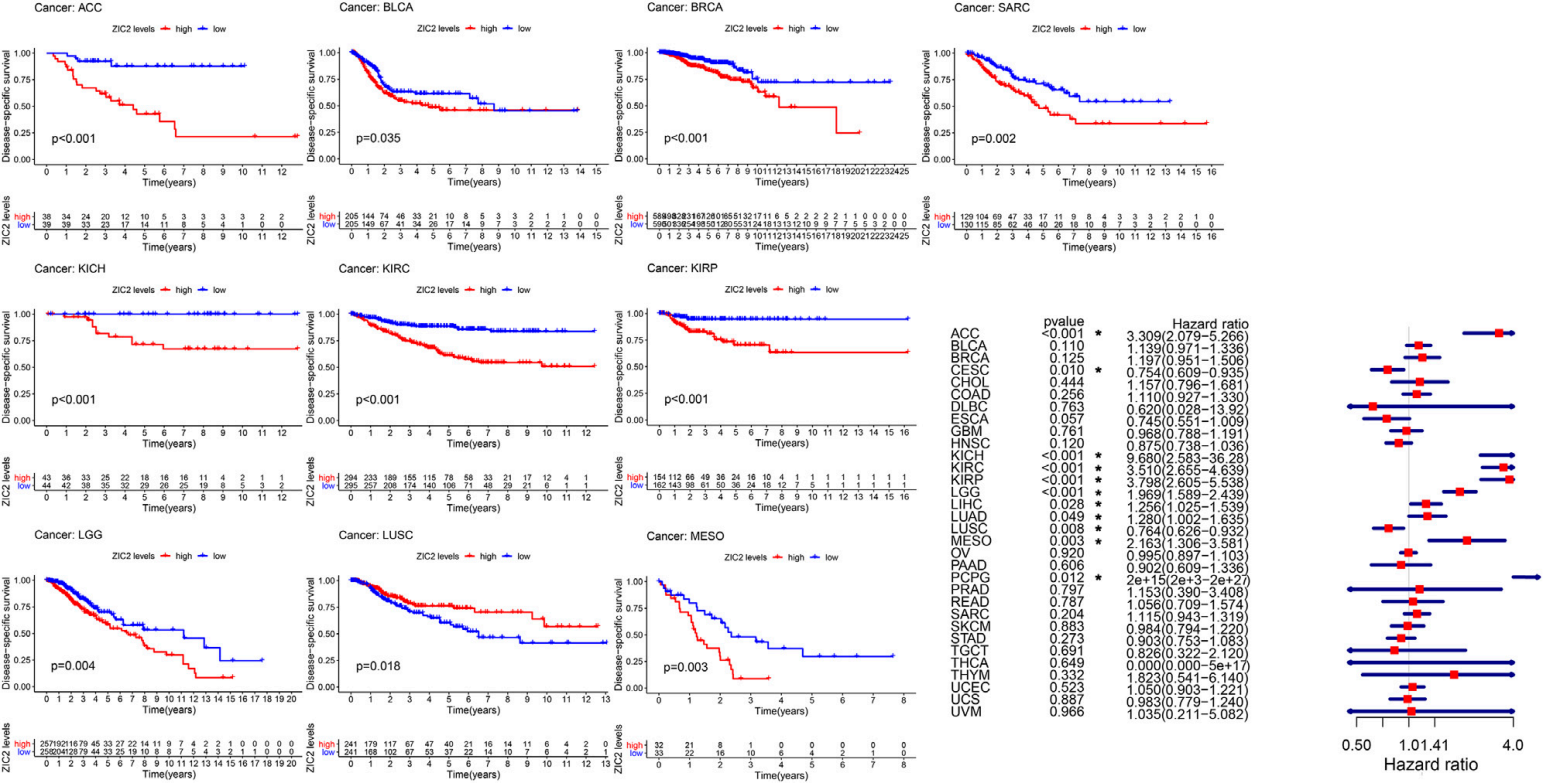
The relationship between ZIC2 expression and DSS in 33 tumors. The survival curves were calculated using K-M methods and the forest plots were calculated using univariate Cox regression. (*p < 0.05)

Considering the possibility of death from non-tumor causes during follow-up, we analyzed the relationship between gene expression and DSS in 33 TCGA tumors. K-M survival analysis showed that low expression of ZIC2 was associated with poor DSS only in LUSC. high ZIC2 expression was associated with poor DSS in nine tumors (ACC, BLCA, BRCA, KICH, KIRC, KIRP, LGG, MESO and SARC). The univariate cox regression results also showed that high expression of ZIC2 was only a protective factor in CESC and LUSC, but was all a risk factor in the nine tumors (ACC, KICH, KIRC, KIRP, LGG, LIHC, LUAD, MESO and PCPG) (Figure 3).

### Correlation of ZIC2 Expression and DFI

**FIGURE 4:**
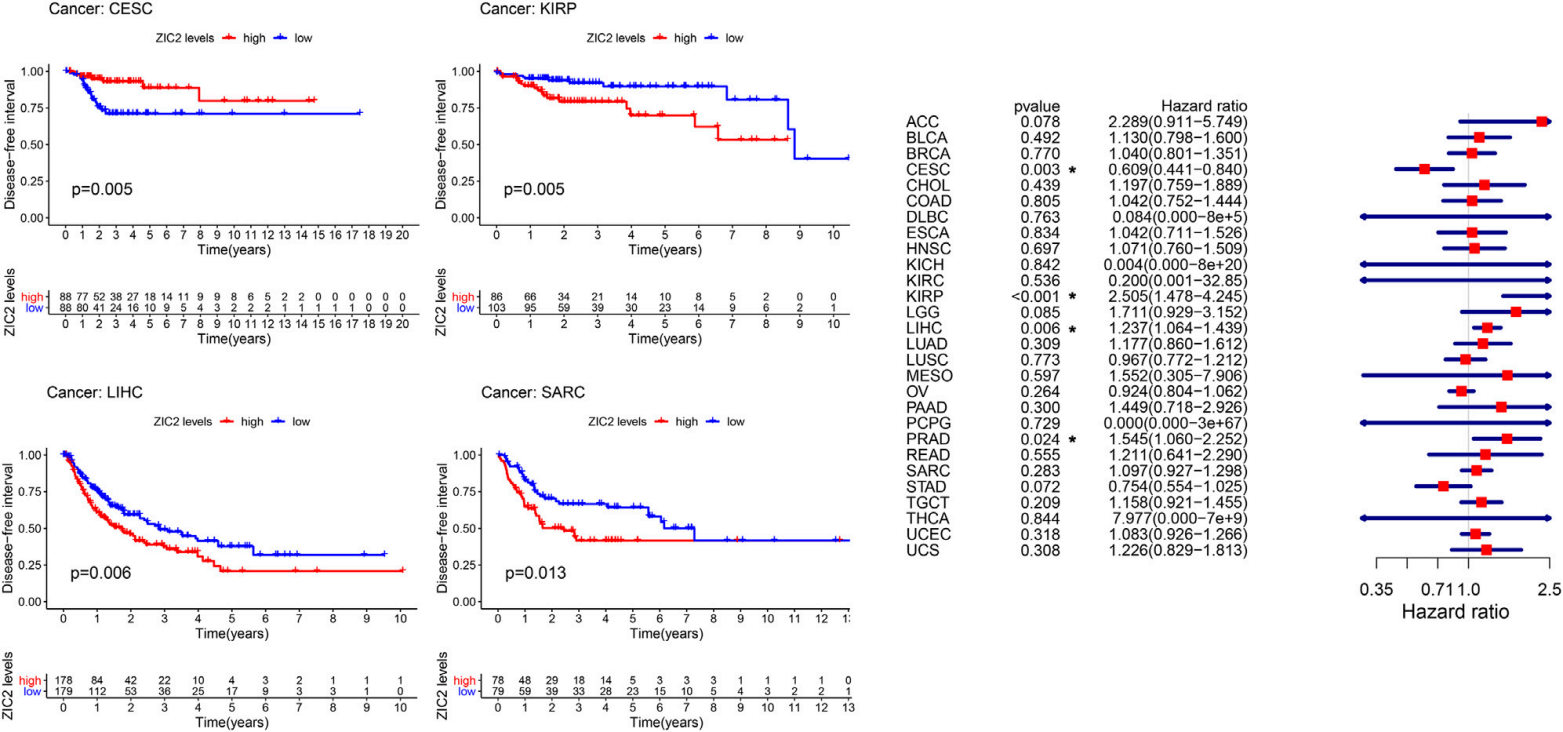
The relationship between ZIC2 expression and DFI in 33 tumors. The survival curves were calculated using K-M methods and the forest plots were calculated using univariate Cox regression. (*p < 0.05)

The same method is used to analyze DFI in 33 TCGA tumors. K-M curve showed that low expression of ZIC2 was associated with poor DSS time in CESC, and high expression of ZIC2 was associated with poor DSS time in the tumor of KIRP, LIHC and SARC. The univariate cox regression results also identified that high expression of ZIC2 was a protective factor in CESC but was all a risk factor in the KIRP, LIHC and PRAD (Figure 4).

### Correlation of ZIC2 Expression and PFI

**FIGURE 5:**
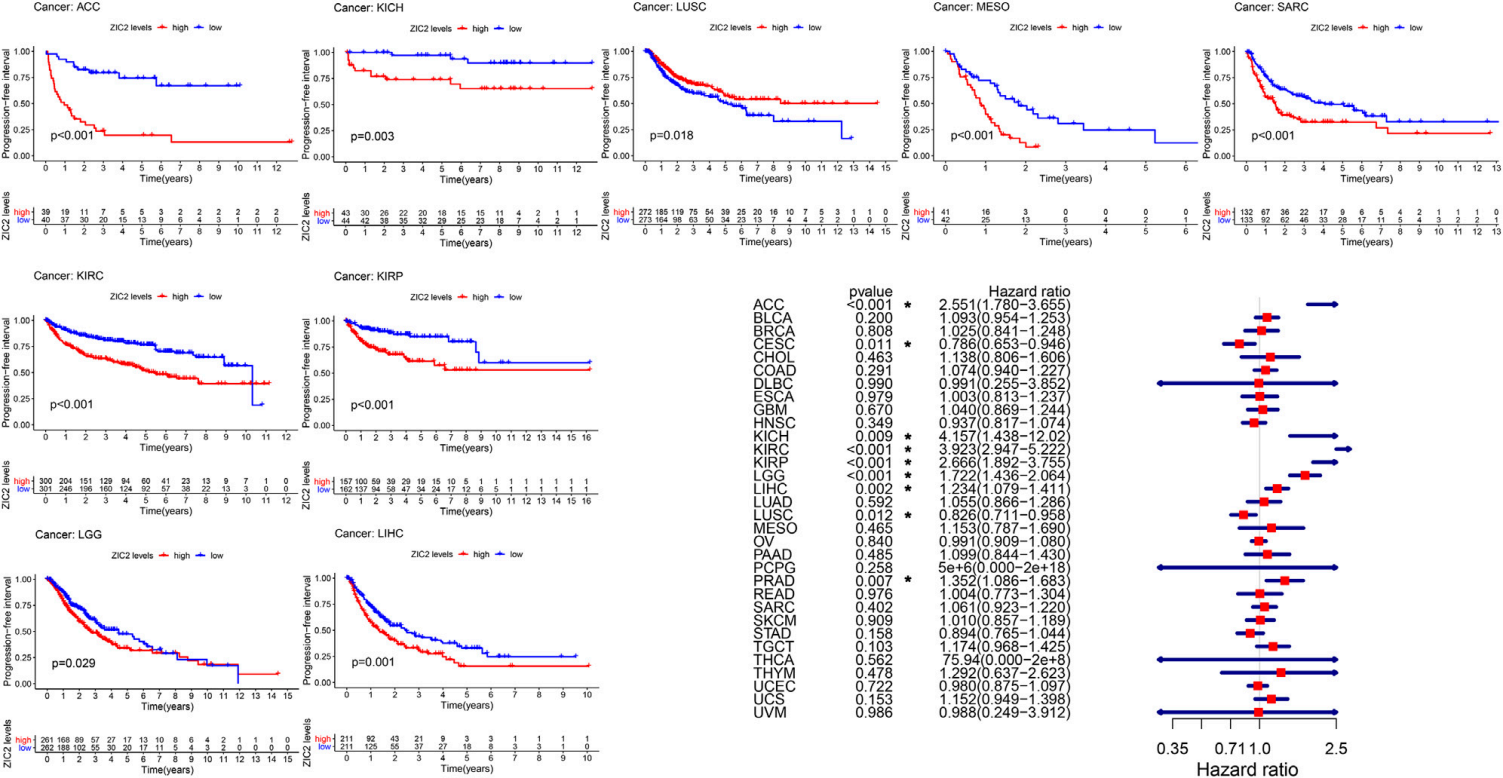
The relationship between ZIC2 expression and PFI in 33 tumors. The survival curves were calculated using K-M methods and the forest plots were calculated using univariate Cox regression. (*p < 0.05)

Finally, we also analyzed the PFI in 33 TCGA tumors. K-M curve that high expression of ZIC2 was associated with poor PFI time in eight types of cancer (ACC, KICH, KIRC, KIRP, LGG, LIHC, MESO, and SARC) and only showed that the low expression of ZIC2 was related to poor PFI time in cancer of LUSC. Univariate cox regression showed similar prognostic results. The high expression of ZIC2 was a protective factor in CESC and LUSC, but was all a risk factor in the ACC, KICH, KIRC, KIRP, LGG, LIHC, and PRAD (Figure 5).

### Validation of the Association of ZIC2 Expression with Prognosis in Other Databases

**FIGURE 6:**
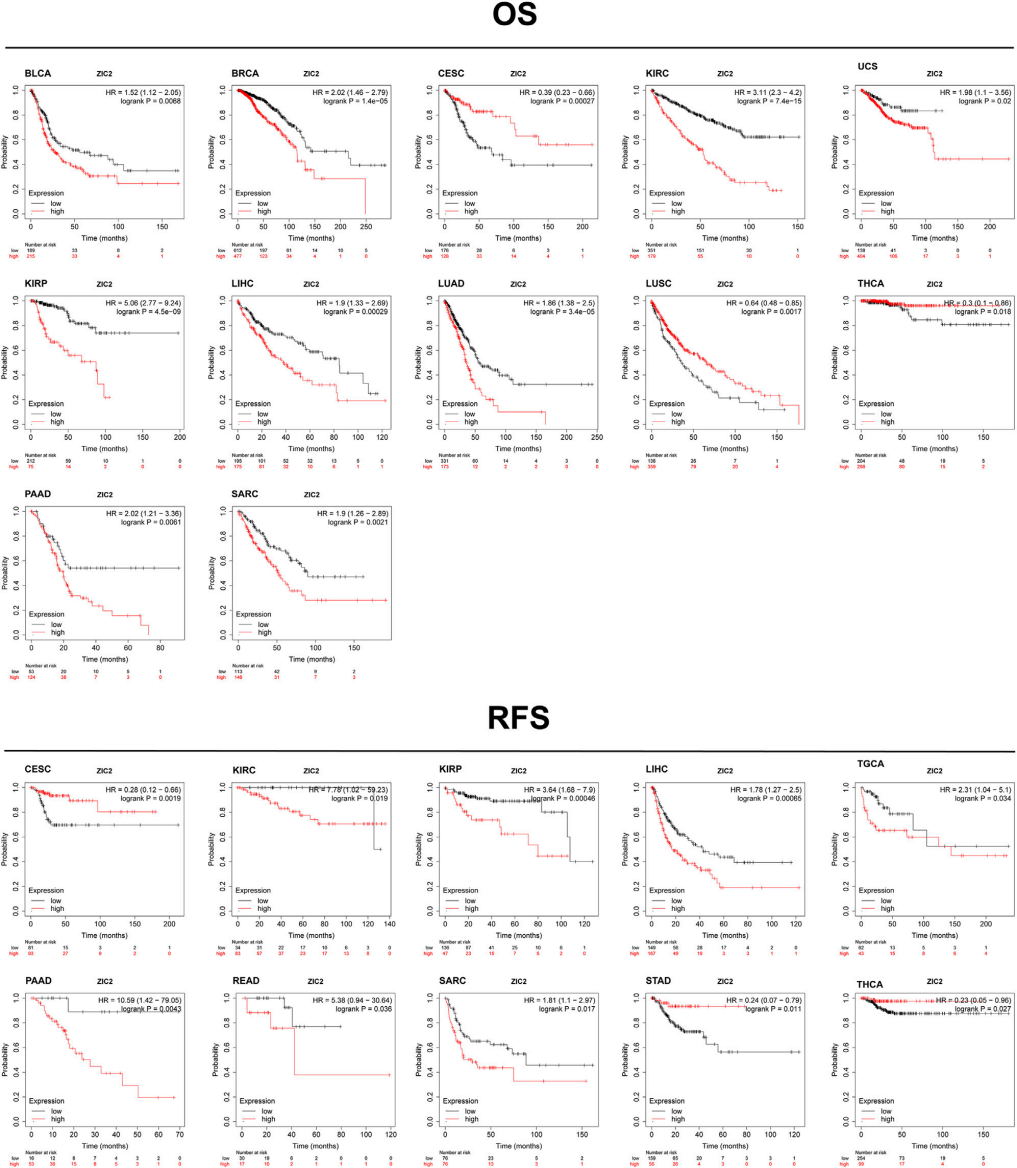
Validation of the relationship between ZIC2 expression and prognosis in Kaplan-Meier Plotter database.

Based on the Kaplan-Meier Plotter database, we next evaluated ZIC2-related OS and RFS. Interestingly, we found that ZIC2 was a detrimental prognostic factor for OS in BLCA, BRCA, KIRC, KIRP, LIHC, LUAD, PAAD and SARC, and only played a prognostic protective role in CESC, LUSC and THCA. With regard to RFS, ZIC2 expression also had a detrimental effect in KIRC, KIRP, LIHC, TGCA, PAAD, READ and SARC, and played a protective prognostic role only in CESC, STAD and THCA (Figure 6). Lastly, we investigated the PrognoScan database mainly incorporating GEO datasets. The results showed that ZIC2 also acted as a risk prognostic factor in bladder, breast and lung cancer Table 1. These results were consistent with the TCGA results, indicating the robustness of the evidence.

**TABLE 1:** ZIC2 gene expression was associated with the prognosis of pan-cancer in PrognoScan.

Dataset	Cancer type	Endpoint	*N*	COX *p*-Value	HR [95% CI]
GSE13507	Bladder cancer	DSS	165	3.46E−05	2.16 [1.50–3.12]
GSE13507	Bladder cancer	OS	165	3.53E−05	1.82 [1.37–2.42]
GSE31210	Lung cancer	RFS	204	0.00039863	1.33 [1.13–1.55]
GSE1378	Breast cancer	RFS	60	0.00207109	1.54 [1.17–2.03]
GSE31210	Lung cancer	OS	204	0.00467843	1.35 [1.10–1.66]
GSE4922-GPL97	Breast cancer	DFS	249	0.0148796	1.24 [1.04–1.48]
GSE9893	Breast cancer	OS	155	0.0169037	1.89 [1.12–3.19]
GSE1379	Breast cancer	RFS	60	0.017022	1.31 [1.05–1.63]
GSE1456-GPL97	Breast cancer	DSS	159	0.0231347	1.46 [1.05–2.03]
GSE1456-GPL97	Breast cancer	RFS	159	0.0258117	1.37 [1.04–1.81]
GSE6532-GPL570	Breast cancer	RFS	87	0.0376658	1.38 [1.02–1.88]
GSE6532-GPL570	Breast cancer	DMFS	87	0.0376658	1.38 [1.02–1.88]

### Correlation of ZIC2 Expression and Clinicopathologic Characteristics

**FIGURE 7:**
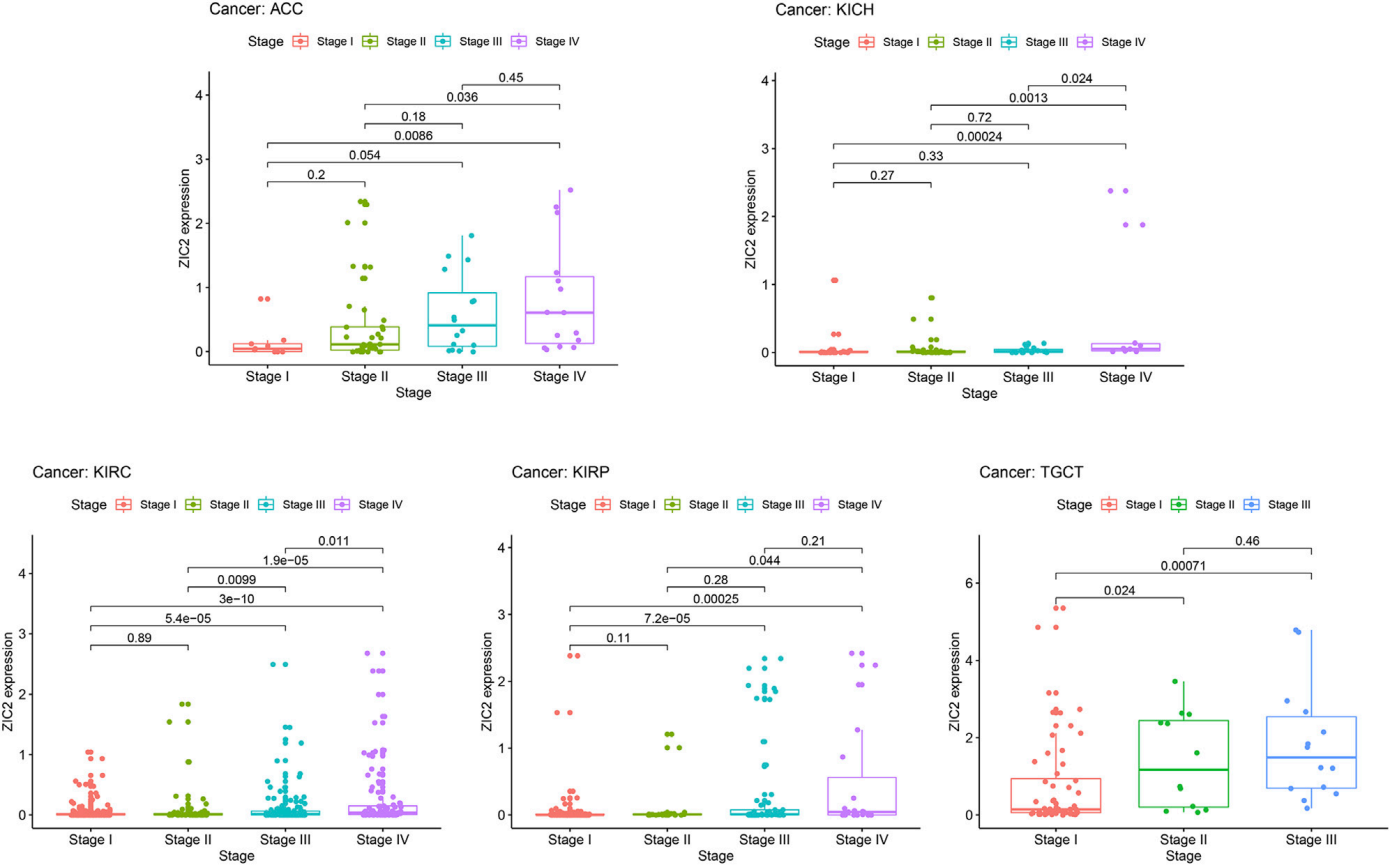
The relationship between ZIC2 expression and clinicopathologic stage in 5 tumors.

In general, late tumor stage in patients is often associated with poor prognosis. We explored the relationship of ZIC2 expression and clinicopathologic stage. The results demonstrated that the ZIC2 mRNA levels in several tumor tissues were significantly different in different clinical stages. With the increase of tumor grade, the expression of ZIC2 also increased, especially in the tumor of ACC, KICH, KIRC, KIRP and TGCT (P < 0.05) (Figure 7).

### Correlation of ZIC2 Expression and TMB, MSL, and Tumor Immune Microenvironment

**FIGURE 8:**
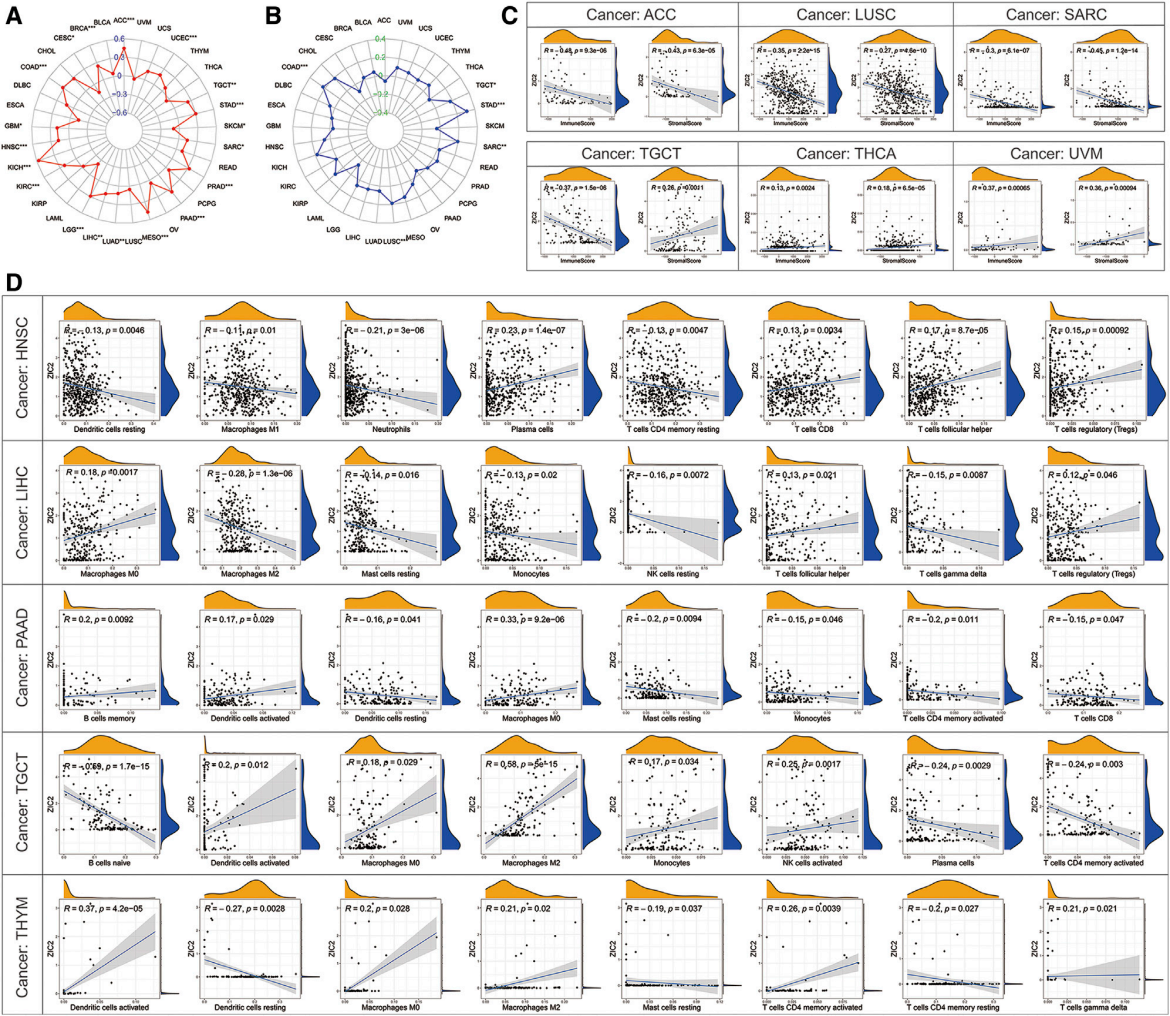
Correlation of ZIC2 expression and TMB, MSL and tumor immune microenvironment. **(A,B)** The radar graphs of correlation of ZIC2 with TMB and MSL. The coordinate value corresponding to each point is the correlation coefficient. (***p < 0.001; **p < 0.01; *p < 0.05) **(C)** Correlation of ZIC2 with immune score and stromal score. **(D)** Correlation of ZIC2 with the content of all immune cells.

TMB represents the density of the distribution of non-synonymous mutations in the protein coding region, which is closely related to tumor prognosis and response to immunotherapy. Here, we calculated the TMB of each sample and then explored its relationship with the expression of ZIC2. We found that ZIC2 expression was significantly associated with TMB in ACC, BRCA, CESC, COAD, GBM, HNSC, KICH, KIRC, LGG, LIHC, LUAD, MESO, PAAD, PRAD, SARC, SKCM, STAD, TGCT and UCEC (Figure 8A). MSI refers to the phenomenon that the length of MS sequence changes due to insertion or deletion mutations during DNA replication, often caused by mismatch repair system (MMRs) functional defects, which is related to the occurrence of cancer and can be used for cancer detection. We also calculated the MSI of each sample and then explored its relationship with the expression of ZIC2. We found that gene expression was significantly correlated with MSI in COAD, LUSC, SARC, STAD and TGCT (Figure 8B). The tumor immune microenvironment has been a key and core direction in tumor research for a long time. It is of great significance for understanding the occurrence, development, and metastasis of tumors, and it also plays an important role in tumor diagnosis, prevention, and prognosis. We calculated the immune score and stromal score of each tumor sample and found there is a significantly correlation between ZIC2 expression and immune / stromal scores. Figure 8C showed the six most significant tumors, showing that ZIC2 expression levels were significantly negatively correlated with immune score and stromal score in SARC, LUSC and ACC, and positively in UVM and THCA. But in TGCT, the ZIC2 expression was negatively correlated with immune score and positively correlated with stromal score. The detailed information is shown in Supplementary Table S2. As an important part of tumor microenvironment, tumor-infiltrating immune cells are related to tumor progression, prognosis and response to immunotherapy. We studied the degree of immune invasion in pan-cancer with different expression levels of ZIC2. Here we presented the five tumors with the strongest correlation (BLCA, LIHC, and LUSC), and the results showed that the expression of ZIC2 was significantly correlated with the level of infiltrating immune cells (Figure 8D). The detailed results of all 33 types of cancer is shown in Supplementary Table S3.

### Co-Expression of ZIC2 with Some Specific Genes

**FIGURE 9:**
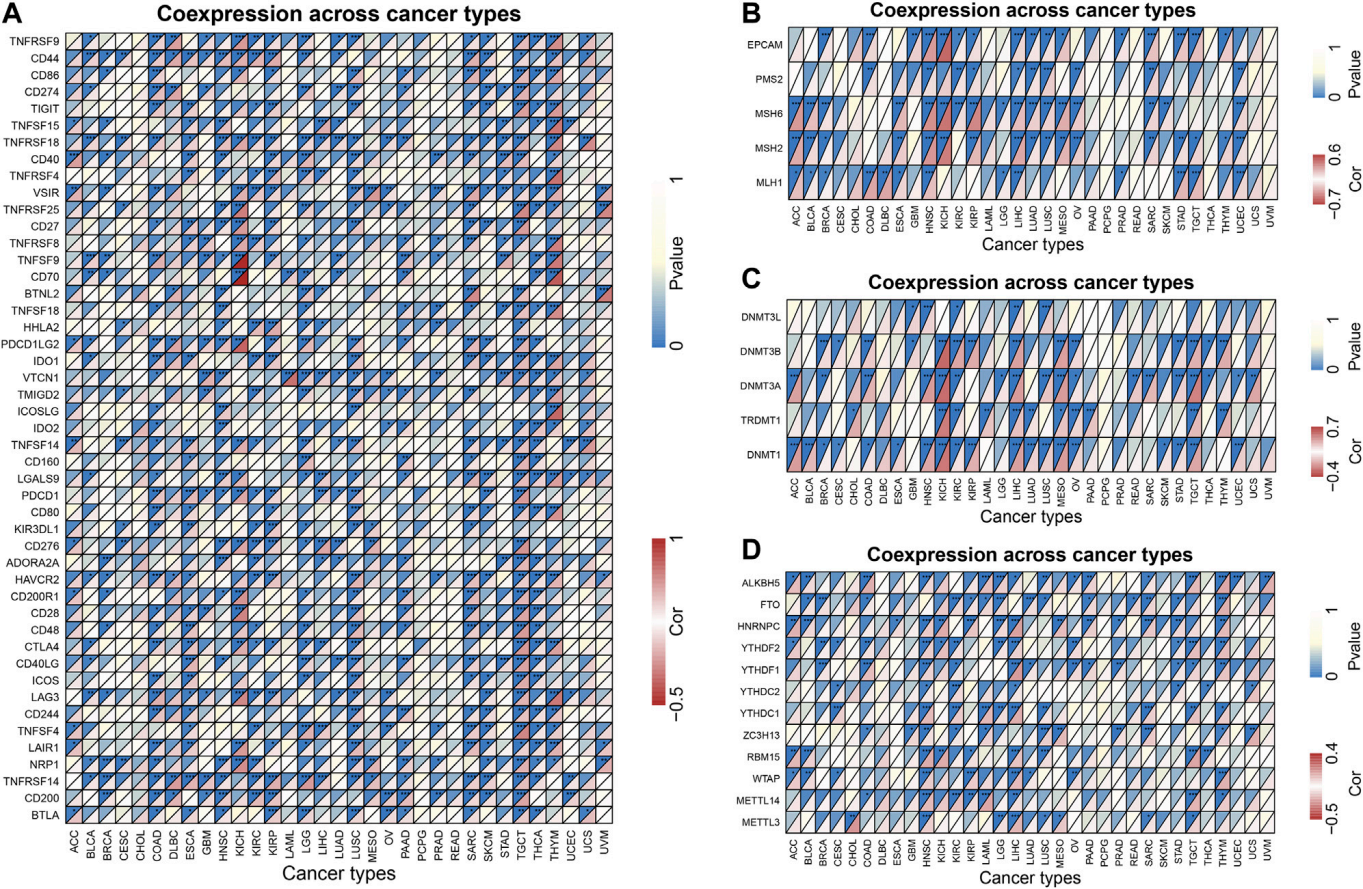
Co-expression of ZIC2 with some specific genes in 33 tumors. **(A)** Co-expression of ZIC2 with immune checkpoint related genes. **(B)** Co-expression of ZIC2 with MMRs genes. **(C)** Co-expression of ZIC2 with DNA methyltransferases. **(D)** Co-expression of ZIC2 with mRNA m6A related genes.

Tumor immunotherapy is a hot topic nowadays. Here, we extracted more than 40 common immune checkpoint genes and analyzed the relationship between the expression of ZIC2 and these immune checkpoint genes. The result showed that the expression of ZIC2 gene was significantly correlated with the expression of many of these immune checkpoint genes (Figure 9A) (Supplementary Table S4). MMRs is an intracellular mismatch repair mechanism, in which the loss of key gene function leads to DNA replication errors leading to the production of higher somatic mutations, which may lead to the development of tumors. We found that five MMRs gene expressions (MLH1, MSH2, MSH6, PMS2, EPCAM) were significantly correlated with ZIC2 expression in multiple tumors (Figure 9B) (Supplementary Table S5). DNA methylation is an epigenetic modification, which plays an important regulatory role in the growth, development, gene expression pattern and genome stability of an individual without changing the DNA sequence. This modification It can be stably transmitted during development and cell proliferation. A large number of studies in recent years have shown that abnormal DNA methylation is closely related to the occurrence and development of tumors and cell carcinogenesis. We found that ZIC2 expression was correlated with the expression of five methyltransferases (DNMT1, TRDMT1, DNMT3A, DNMT3B, DNMT3L) in multiple tumors (Figure 9C) (Supplementary Table S6). m6A (6-methyladenine) is the most common type of RNA methylation modification on mRNA, which plays an important role in tumor development. The modification level of m6A is dynamically regulated by methyltransferase (six genes), binding protein (five genes) and demethylase (two genes). We also found that ZIC2 expression was shown to be significantly associated with the expression of these 13 genes (Figure 9D) (Supplementary Table S7).

### GSEA Analysis

**FIGURE 10:**
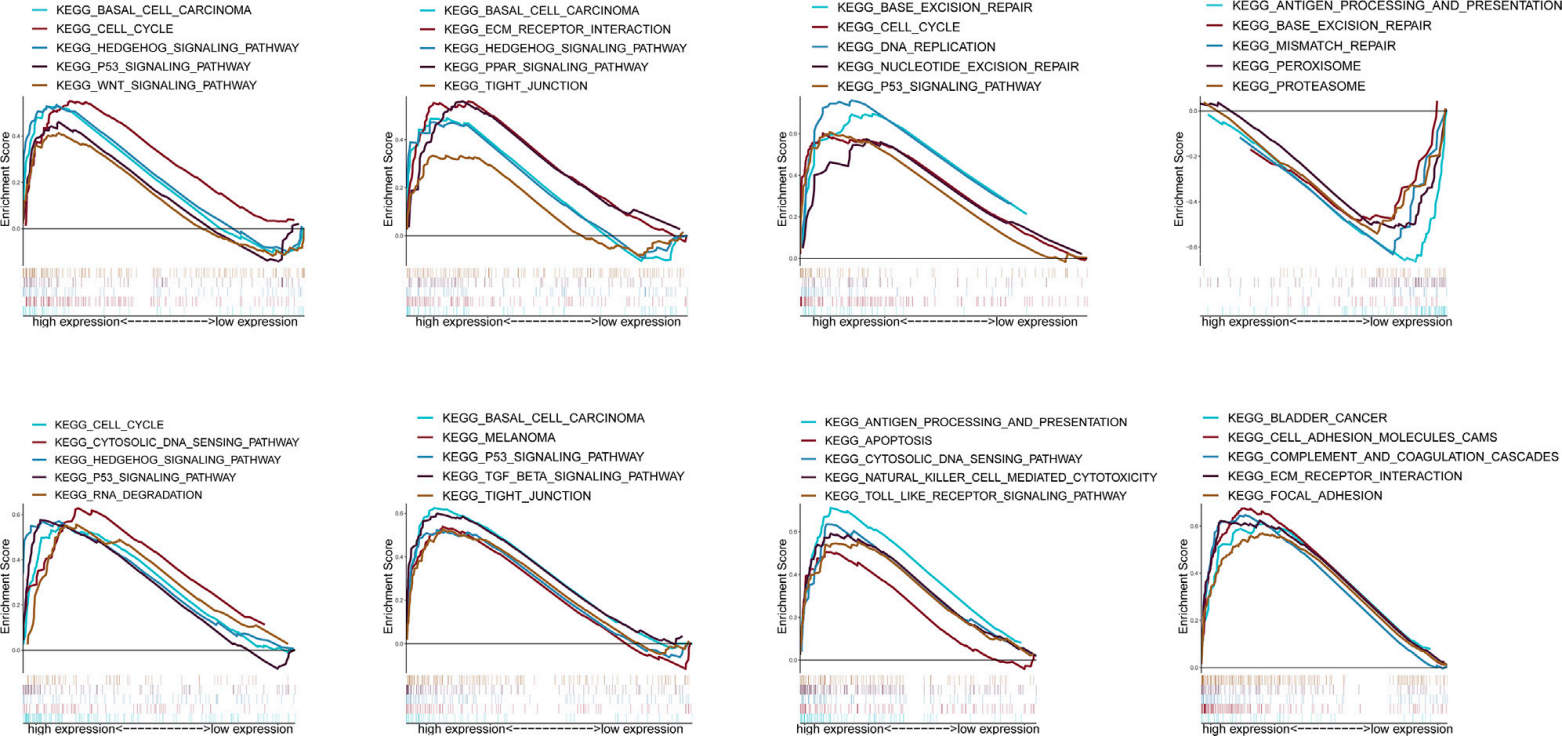
Enrichment plots from GSEA.

According to the median expression of ZIC2, all samples were divided into high expression group and low expression group. GSEA was used to analyze which KEGG pathways were enriched in high or low expression groups. The results revealed that multiple tumor- and immune-related pathways were differentially enriched in ZIC2 high or low expression phenotype in a variety of tumors including complement and coagulation cascades, P53 signaling pathway, basal cell carcinoma, PPAR signaling pathway, tight junction, etc (Figure 10).

## Discussion

ZIC2 protein consists of 532 amino acids, including an N-terminal domain involved in transcriptional regulation, five C2H2 zinc finger repeats as DNA binding domain, and a C-terminal domain (Mizugishi et al., 2001; Mizugishi et al., 2004). ZIC2 was first discovered to be at important factor in human embryos, the brain and nervous system, vision, myogenesis, and the establishment of the left and right axes (Nagai et al., 2000; Aruga et al., 2002; Herrera et al., 2003; Pan et al., 2011; Dykes et al., 2018). In recent years, ZIC2 has attracted extensive attention in the field of cancer research. A lot of studies have reported that the overexpression of ZIC2 was significantly correlated with tumor migration, metastasis, angiogenesis or poor prognosis in patients, and it could be a potential tumor marker or therapeutic target. However, these studies of ZIC2 have focused on a single or limited number of cancer types with small sample size. Therefore, the significance of clinical guidance is often underpowered. In addition, its expression and prognostic significance in pan-cancer are unclear.

In present study, we comprehensively conducted this systematic analysis of ZIC2 across 33 tumor types. First, we compared the expression of ZIC2 in 33 tumors and their corresponding normal tissues, and found that ZIC2 was differentially expressed in up to 20 tumors, and most of them were highly expressed in tumors compared with normal tissues. Second, we explored whether ZIC2 expression is related to OS, DSS, DFI and PFI. We found that in most tumors, high expression of ZIC2 was a risk factor and associated with poor OS, DSS, DFI and PFI. Third, We also found that the high expression of ZIC2 was significantly correlated with the late stage in various tumors. in multiple tumors. Fourth, some studies have confirmed that TMB has been considered a potential predictive biomarker in immunotherapy (Samstein et al., 2019), and could be used as a prognostic biomarker (Tang et al., 2020). Similarly, MSI could also be used as a prognostic biomarker to guide the decision-making of adjuvant chemotherapy, targeted therapy and immunotherapy for tumors (Zhang et al., 2016; Shen et al., 2017a). Previous studies have also shown that immune infiltration is a crucial factor in determining the responsiveness and prognosis of cancer (McGranahan et al., 2016). Given that, we explored the correlation of ZIC2 expression and TMB, MSL and tumor immune microenvironment. Our study demonstrated surprisingly strong associations between ZIC2 expression and TMB, MSI, and immune invasion in many tumors. Fifth, we also explored the co-expression of ZIC2 with immune checkpoint related genes, MMRs gene, DNA methyltransferase gene and M6A RNA methylation related genes. All of the four target genes discussed above were related to tumor development and immunotherapy. We also confirmed that there was a significant co-expression relationship between ZIC2 expression and the above four type genes in multiple tumors. In brief, these results suggested that ZIC2 may have crucial effect to the tumorigenesis by influencing TMB and MSI, recruiting and regulating of cancer immune infiltrating cells and regulating immune checkpoint, MMRs, DNA methyltransferase gene and M6A RNA methylation ultimately affecting the patient prognosis. Taken together, these findings strongly indicated that ZIC2 can be used as a biomarker to judge the prognosis of various cancers. We finally used GSEA to try to explore its potential pathway mechanism. As it turned out, the results showed that multiple tumor- or immune-related pathways were enriched.

Our summary results were consistent with current findings from other clinical and basic studies. In nasopharyngeal carcinoma, the increased expression of ZIC2 leads to increased proliferation, invasion, lymphangiogenesis and lymphatic metastasis of tumor cells (Shen et al., 2017b; Yu et al., 2020), and was an independent prognostic factor of OS and DFS (Yi et al., 2018). ZIC2 was also highly expressed in breast cancer tissues, promoting the proliferation, migration and invasion of breast cancer cells (Zhang et al., 2019). Jiang et al. (2019) found that ZIC2 was highly expressed in prostate cancer and can promote cell migration, invasion, angiogenesis and tumorigenesis, while reducing cell apoptosis. Similarly, high expression of ZIC2 was also found in acute myeloid leukemia (AML), and it can promote proliferation and reduce apoptosis of AML cells (Chen et al., 2019). ZIC2 was also highly expressed in osteosarcoma cells and tissues, and its overexpression promoted viability, invasion of osteosarcoma cells (Huang and Jin, 2018). High expression of ZIC2 was also shown in bladder cancer, which can regulate the vitality and apoptosis of bladder cancer cells (Wang et al., 2017). One study has found that ZIC2 expression gradually increased from normal tissues and liver cancer tissues to metastatic tissues, which can promote the proliferation and migration of liver cancer cells in vitro and promote growth and metastasis of solid tumors in vivo (Lu et al., 2017). Another study showed that ZIC2 was highly expressed in liver cancer stem cells and drove self-renewal of liver cancer stem cells. In pancreatic ductal adenocarcinoma (PDAC) cell, ZIC2 is indispensable in the regulation of PDAC cell apoptosis. Knockdown of ZIC2 induced apoptosis of PDAC cell; in contrast, overexpression of ZIC2 promoted the cell proliferation (Inaguma et al., 2015). Epithelial ovarian tumor, as one of the most fatal malignant tumors in women, can be divided into malignant form (MAL) and low malignant potential (LMP). Overexpression of ZIC2 increased the growth rate and lesion formation and stimulated the formation of anchor independent colonies; conversely, down-regulation of ZIC2 decreased the growth rate of MAL cell line (Marchini et al., 2012). An analysis of 18 sporadic medulloblastomas revealed that ZIC2 is a frequently methylated gene with adverse clinical outcomes. (Pfister et al., 2007). All the above research results can be used as the verification of our bioinformatics results. and it may indicate that ZIC2 may act as an oncogenic driver in human cancer.

Although our results showed that ZIC2 has strong clinical predictive significance in pan-cancer, some conflicting results still existed. On the basis of our current research and that of others so far, high expression of ZIC2 in most tumors has been observed to be an oncogene and a risk factor for poor prognosis of tumors. However, our founding indicated that ZIC2 was actually a protective factor for DSS and PFI in LUSC. We have not found any study on the relationship between ZIC2 and lung cancer by reviewing the current research literature. Besides, we found that the high expression of ZIC2 in CESC, but the high expression of ZIC2 showed a protective factor for the prognosis of the disease. Paradoxically, Wang et al. found the transcription and translation levels of ZIC2 was high in cervical cancer cell, which can promote angiogenesis and cell migration and invasion (Wang et al., 2018). Moreover, function gain and loss analysis of ZIC2 showed that it could improve the Hh signal transduction activity, cell proliferation and anchorage-independent growth ability in cervical cancer cells (Chan et al., 2011). For the above contradictory results, we can find the reasons from the following aspects. First, although the TCGA database is rich in data resources, it contains a small sample size of normal tissues, which may lead to the occurrence of confounding bias due to different baseline levels including clinical background factors between the two sides of the comparison. Second, it is possible that the expression of ZIC2 may change in the early stage of the occurrence of the above two cancers, but the change may be not obvious during the development of the cancer. Thirdly, the high expression of ZIC2 may be related to the occurrence of cancer, while the low expression may be related to the development of cancer, which indicates that the gene ZIC2 does not play a role alone and may be passively and dynamically regulated in the process of tumor development. Finally, we should also recognize that there is significant heterogeneity between different tumors and even between different populations of the same tumor, which requires more comprehensive studies in the future to reveal the truth.

Our research also had certain limitations. Firstly, this study had the relatively small sample size in some rare tumor types and may lead to potentially inaccurate or unstable results. Secondly, some cancer species lack normal tissues as controls, including ACC, DLBC, LAML, LGG, MESO, OV, TGCT, UCS and UVM. Finally, all of our results were based on public database and were not verified by experiments.

Careful analysis of this study will find some interesting findings and provide future research directions. For example, the consistent high expression of ZIC2 in renal tumors significantly indicates poor prognosis, and this trend exists in any pathological type (KICH, KIRC and KIRP). We are already conducting laboratory experiments on cell lines to explore their effects on the ability of on cell proliferation, invasion or metastasis by promoting or inhibiting its expression. As a potential diagnostic or prognostic marker, there is still a long way to go for its real clinical application.

## Conclusion

In conclusion, our analysis suggested that ZIC2 displayed high expression in multiple tumors and was significantly associated with tumor progress and patient survival. We demonstrated some potential mechanisms by which ZIC2 promoted tumor progression, and further suggested that ZIC2 may act as an oncogene with a strong effect in the processes of tumorigenesis and progression. However, large amount of verification experiment was still needed in the future.

## Data Availability

Publicly available datasets were analyzed in this study. This data can be found here: http://xena.ucsc.edu, http://www.oncmine.org, https://kmplot.com/ analysis and http://www.prognoscan.org.

## Glossary

ACC: Adrenocortical carcinoma;
BLCA: Bladder Urothelial Carcinoma;
BRCA: Breast invasive carcinoma;
CESC: Cervical squamous cell carcinoma and endocervical adenocarcinoma;
CHOL: Cholangiocarcinoma;
COAD: Colon adenocarcinoma;
DFI: Disease-Free Interval;
DLBC: Lymphoid Neoplasm Diffuse Large B-cell Lymphoma;
DSS: Disease-Specific Survival;
ESCA: Esophageal carcinoma;
GBM: Glioblastoma multiforme;
GEO: Gene Expression Omnibus;
GSEA: Gene set enrichment analysis;
HNSC: Head and Neck squamous cell carcinoma;
KEGG: Kyoto Encyclopedia of Genes and Genomes;
KICH: Kidney Chromophobe;
KIRC: Kidney renal clear cell carcinoma;
KIRP: Kidney renal papillary cell carcinoma;
K-M: Kaplan-Meier curves;
LAML: Acute Myeloid Leukemia;
LGG: Brain Lower Grade Glioma;
LIHC: Liver hepatocellular carcinoma;
LUAD: Lung adenocarcinoma;
LUSC: Lung squamous cell carcinoma;
MESO: Mesothelioma;
MMRs: Mismatch repair system;
MSI: Microsatellite instability;
OS: Overall Survival;
OV: Ovarian serous cystadenocarcinoma;
PAAD: Pancreatic adenocarcinoma;
PCPG: Pheochromocytoma and Paraganglioma;
PFI: Progression-Free Interval;
PRAD: Prostate adenocarcinoma;
READ: Rectum adenocarcinoma;
SARC: Sarcoma;
SKCM: Skin Cutaneous Melanoma;
STAD: Stomach adenocarcinoma;
TMB: Tumor mutation burden;
TCGA: The Cancer Genome Atlas;
TGCT: Testicular Germ Cell Tumors;
THCA: Thyroid carcinoma;
THYM: Thymoma;
UCEC: Uterine Corpus Endometrial Carcinoma;
UCS: Uterine Carcinosarcoma;
UVM: Uveal Melanoma;
ZIC2: Zic family member 2.
